# The Multifaceted Role of *IL-35* in Periodontal Disease and Beyond: From Genetic Polymorphisms to Biomarker Potential

**DOI:** 10.3390/genes16080891

**Published:** 2025-07-28

**Authors:** Zdravka Pashova-Tasseva, Antoaneta Mlachkova, Kamen Kotsilkov, Hristina Maynalovska

**Affiliations:** Department of Periodontology, Faculty of Dental Medicine, Medical University of Sofia, 1000 Sofia, Bulgaria; a.mlachkova@fdm.mu-sofia.bg (A.M.); k.kotsilkov@fdm.mu-sofia.bg (K.K.); h.maynalovska@fdm.mu-sofia.bg (H.M.)

**Keywords:** *interleukin-35*, periodontitis, cytokines, non-surgical periodontal therapy, biomarkers, genetic polymorphisms, inflammation, immune regulation

## Abstract

Periodontitis is a prevalent chronic inflammatory disease with complex etiopathogenesis involving microbial dysbiosis, host immune response, environmental factors, and genetic susceptibility. Among the cytokines implicated in periodontal immunoregulation, *interleukin-35* (*IL-35*) has emerged as a novel anti-inflammatory mediator with potential diagnostic and therapeutic relevance. This narrative review evaluates the role of *IL-35* in periodontal disease by exploring its local and systemic expression, response to non-surgical periodontal therapy (NSPT), and association with clinical disease severity. Additionally, current evidence regarding *IL-35* gene polymorphisms and their potential contribution to individual susceptibility and disease progression, as well as their relevance in related systemic conditions, is assessed. A comprehensive review and synthesis of recent clinical and experimental studies were conducted, focusing on *IL-35* levels in saliva, serum, and gingival crevicular fluid (GCF) among patients with healthy periodontium, gingivitis, and various stages of periodontitis, both before and after NSPT. Emphasis was placed on longitudinal studies evaluating *IL-35* dynamics in correlation with periodontal parameters, as well as genetic association studies investigating *IL-12A* and *EBI3* gene polymorphisms. *IL-35* levels were generally found to be higher in healthy individuals and reduced in periodontitis patients, indicating a possible protective role in maintaining periodontal homeostasis. Following NSPT, *IL-35* levels significantly increased, corresponding with clinical improvement and reduced inflammatory burden. Genetic studies revealed variable associations between *IL-35* polymorphisms and susceptibility to periodontitis and related systemic conditions, although further research is needed for validation. *IL-35* appears to function as a modulator of immune resolution in periodontal disease, with potential utility as a non-invasive biomarker for disease activity and therapeutic response. Its upregulation during periodontal healing supports its role in promoting tissue stabilization. The integration of cytokine profiling and genetic screening may enhance personalized risk assessment and targeted interventions in periodontal care.

## 1. Introduction

Periodontitis is a highly prevalent chronic inflammatory disease affecting approximately 10–15% of adults worldwide, with significant public health implications [1]. It spans all geographic regions and socio-economic groups and is a major contributor to the global burden of oral diseases. As a leading cause of tooth loss and edentulism in adults, periodontitis compromises mastication, nutrition, esthetics, and overall quality of life. Its systemic consequences and well-established associations with chronic conditions such as cardiovascular disease, diabetes mellitus, and adverse pregnancy outcomes further underscore its medical and societal relevance [2,3].

The etiology of periodontitis is multifactorial, involving complex interactions between oral microbial communities, host immune responses, and environmental and behavioral influences. While microbial colonization initiates the disease, it is the host’s dysregulated inflammatory and immune reaction that drives tissue destruction and disease progression. These responses are shaped by both non-modifiable factors—including age, sex, ethnicity, and genetic predisposition—and modifiable ones, such as inadequate oral hygiene, colonization by specific periodontal pathogens (e.g., *Porphyromonas gingivalis*, *Tannerella forsythia*, and *Treponema denticola*), and lifestyle behaviors like smoking, one of the most potent environmental risk factors [4]. Systemic conditions including diabetes mellitus, obesity, metabolic syndrome, and osteoporosis further exacerbate periodontal inflammation. Additionally, psychosocial stress, low socio-economic status, and reduced access to dental care influence both susceptibility and treatment outcomes [4].

A critical aspect of disease progression is the transition from a symbiotic to a dysbiotic oral microbiome, characterized by the proliferation of pathogenic Gram-negative anaerobes. This microbial shift activates a host-mediated immune-inflammatory cascade that leads to destruction of the periodontal ligament, alveolar bone, and connective tissue attachment—making periodontitis a classical example of chronic inflammation driven by polymicrobial synergy and dysbiosis [5].

Further complicating this process is individual variability in immune response, influenced not only by environmental exposures but also by genetic and epigenetic factors. A subset of individuals demonstrates an exaggerated or dysregulated immune reaction to common microbial challenges, which has directed scientific focus toward the immunogenetic underpinnings of periodontitis, including cytokine expression profiles, gene polymorphisms, and the interplay between pro- and anti-inflammatory mediators [4,5].

Within this immunological framework, the study of novel cytokines—especially those with regulatory or suppressive properties—has gained momentum. Of particular interest is *interleukin-35* (*IL-35*), a relatively recent addition to the *interleukin-12* cytokine family. *IL-35* has been proposed as a key anti-inflammatory and immunomodulatory molecule with potential roles in immune tolerance, inflammation resolution, and tissue protection. Investigating *IL-35* in the context of periodontitis may provide critical insights into the mechanisms of disease susceptibility, progression, and therapeutic response, particularly as research shifts toward biomarker-guided, individualized approaches in periodontal diagnostics and treatment planning [6].

## 2. Periodontitis

### 2.1. Etiology, Pathogenesis, and Disease Susceptibility

In addition to microbial and environmental influences, genetic and epigenetic determinants critically shape the clinical course of periodontitis. Among environmental risk factors, tobacco smoking is particularly significant, consistently identified as a leading cause of preventable disease and mortality worldwide [6]. Smoking profoundly affects the periodontium, including immune modulation, impaired wound healing, and microbial changes [7].

Periodontitis has gained renewed attention due to its growing social and economic impact. Epidemiological data estimate that 10–15% of the global population is affected [8], with severe forms ranking as the sixth most prevalent disease worldwide—impacting 11.2% or approximately 743 million people. The global prevalence of severe periodontitis nearly doubled between 1990 and 2010 [9].

Left untreated, periodontitis causes tooth loss and edentulism, impairing mastication, nutrition, appearance, and often leading to psychological and social withdrawal. Although more common in older adults, increasing cases in those aged 30–40 suggest an epidemiological shift. Periodontitis is also associated with systemic diseases, as confirmed by multiple systematic reviews, prompting investigations into its mechanisms. Recent research emphasizes individual susceptibility, which varies markedly across populations. A small proportion—5–15%—exhibits heightened susceptibility due to genetic, immunologic, and behavioral factors [10,11,12,13,14,15].

Pathogenetically, periodontitis is mediated by a dysregulated host inflammatory response to a pathogenic biofilm. While microbial agents initiate disease, its progression and severity are largely determined by host immune mechanisms. A key factor is the imbalance between pro- and anti-inflammatory cytokines, contributing to connective tissue breakdown and bone loss [16]. Research has focused on cytokine dysregulation and immune mediators in disease progression. Although pathogenic bacteria are essential, host susceptibility—shaped by genetic, environmental, and immune factors—ultimately governs outcomes. This concept remains central to periodontal research, including responses to microbial insults and therapeutic interventions such as orthodontics [16]. Studying these mechanisms supports improved risk stratification, prevention, and personalized treatment. The disease is tightly linked to the host’s inflammatory response, amplified by persistent pathogens like *Porphyromonas gingivalis*, *Prevotella intermedia*, *Treponema denticola*, and *Tannerella forsythia*, which release virulence factors sustaining chronic inflammation and tissue disruption [16].

This inflammatory state is marked by elevated pro-inflammatory cytokines and suppressed anti-inflammatory responses, creating a destructive environment in periodontal tissues. This leads to degradation of connective tissue and alveolar bone—key features of advanced disease. Importantly, the effects of inflammation extend beyond the local site, with increasing evidence of systemic repercussions [16]. The immune response involves upregulation of pro-inflammatory mediators like *IL-1β*, *TNF-α*, and *IL-6*, promoting immune cell recruitment, MMP activation, and osteoclastic activity. Current research focuses on cytokine regulation and the balance between inflammatory mediators and inhibitors such as *IL-1Ra* and TIMPs. This has supported the development of cytokine-targeted therapies to modulate the host response and preserve tissue [16].

Specific pro-inflammatory cytokines are now recognized as central in periodontal disease pathogenesis. Nevertheless, individual susceptibility remains a key theme in periodontal research [16]. This is partly genetically determined and reflected in variable host responses. Expanding knowledge of genetic predisposition to chronic and inflammatory diseases opens opportunities for precision diagnostics and personalized therapies. In periodontitis, identifying genetically susceptible individuals may enable early risk assessment and preventive strategies, improve diagnostic accuracy, and enable personalized treatment planning.

### 2.2. Genetic Basis of Periodontitis

The pathogenesis of multifactorial diseases involves overlapping biological mechanisms that lead to a common clinical phenotype. Periodontitis exemplifies this complexity and is widely recognized as a polygenic condition, with multiple polymorphic genes influencing disease susceptibility [16]. These variants, often located in regulatory or coding regions, modulate immune and inflammatory responses, impacting disease risk and severity. Many genetic loci may be involved, but their expression and phenotypic outcomes vary across populations [16]. This underscores the influence of environmental modifiers—such as lifestyle, oral hygiene, microbial exposure, and systemic health—which interact with genetic predispositions. Such gene–environment interplay contributes to the heterogeneity seen in clinical presentations [16].

A key focus in periodontal genomics is the analysis of single-nucleotide polymorphisms (SNPs), the most common form of genetic variation. These SNPs can affect protein function or gene expression, particularly in immune-related pathways. For example, polymorphisms in genes encoding cytokines, receptors, metabolic enzymes, and pattern recognition receptors may alter inflammatory signaling and disease susceptibility. Not all SNPs are harmful, some are neutral or may offer protective effects [16].

In periodontitis, particular focus has been placed on SNPs in genes regulating key pro-inflammatory cytokines—such as *IL-1*, *IL-6*, and *TNF-α*—as well as those involved in Toll-like receptor (TLR) signaling and matrix metalloproteinase (MMP) activity. These variations may affect host responses to subgingival biofilms, influencing periodontal inflammation and tissue destruction [16].

Genetic association studies assess the relationship between genotype and phenotype by comparing allele frequencies in affected individuals and healthy controls. When an allele is more frequent in the disease group, the associated SNP is inferred to confer increased susceptibility [16,17]. These findings support the identification of genetic biomarkers for risk assessment, early diagnosis, and targeted therapies. A growing body of literature confirms strong associations between SNPs and both periodontitis susceptibility and clinical severity. Notably, SNPs in genes encoding immune regulatory molecules, especially cytokines and their receptors, modulate inflammatory responses and influence disease onset, progression, and severity [1,18,19].

## 3. Interleukin 35

**Figure 1:**
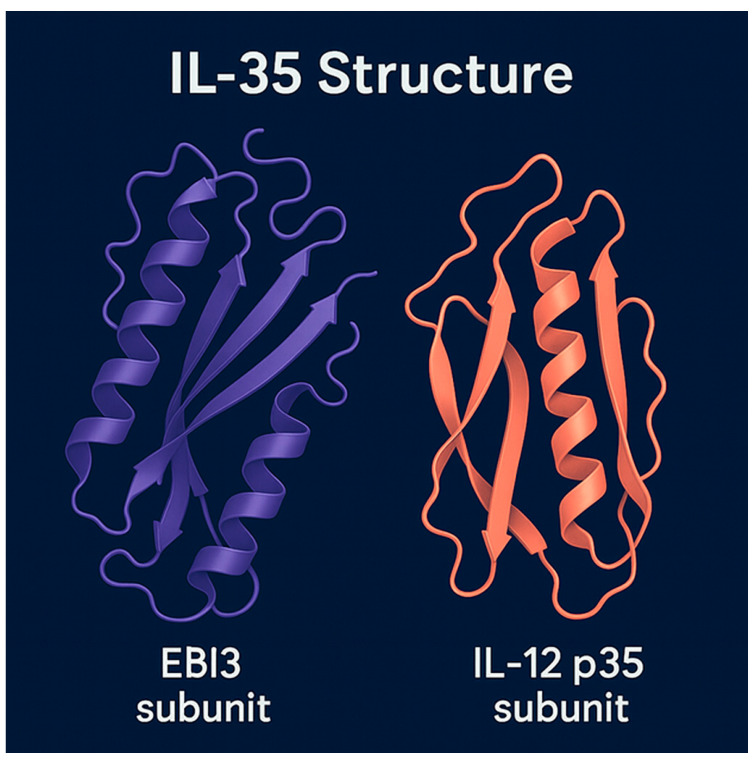
Structural composition of *IL-35*: *Ebi3* and IL-12p35 subunits.

Among the expanding array of cytokines under investigation, *interleukin-35* (*IL-35*) has emerged as a cytokine of considerable interest since the early 21st century, particularly due to its immunoregulatory potential and its proposed role in the pathogenesis and clinical modulation of chronic inflammatory disorders, including periodontitis. Like other members of the *interleukin-12* (*IL-12*) cytokine family, *IL-35* is a heterodimeric molecule composed of two distinct subunits: Epstein–Barr virus-induced gene 3 (*EBI3*) and the IL-12p35 subunit, which it shares with IL-12 (Figure 1) [20].

The IL-12 cytokine family comprises structurally related heterodimers formed by various combinations of five subunits—*p19*, *p28*, *p35*, *p40*, and *EBI3*. These pairs form biologically active cytokines with distinct immunological functions, including IL-12 (p35/p40), IL-23 (p19/p40), IL-27 (p28/*EBI3*), and *IL-35* (p35/*EBI3*). While most family members promote pro-inflammatory pathways and support T helper cell differentiation, *IL-35* is unique in its predominantly suppressive profile, functioning as an anti-inflammatory cytokine [21].

*IL-35* is primarily produced by immunosuppressive cell subsets such as regulatory B cells (Bregs), CD4^+^ regulatory T cells (Tregs), and CD8^+^ regulatory T cells. It exerts potent inhibitory effects on the immune system, particularly by suppressing the proliferation and cytokine production of pro-inflammatory T helper 17 (Th17) cells, a subset implicated in the pathogenesis of periodontitis and other autoimmune disorders [21]. By downregulating IL-17 expression and other pro-inflammatory mediators, *IL-35* contributes to the resolution of inflammation and promotes tissue protection [22,23]. Moreover, *IL-35* induces a unique population of regulatory T cells, termed *IL-35*-induced regulatory T cells (iTr35), which themselves secrete *IL-35*, thereby amplifying the cytokine’s immunosuppressive effects [23,24,25,26,27].

Polymorphisms in the genes encoding the *IL-35* subunits—*EBI3* and IL-12p35—may affect cytokine expression levels, structural integrity, or receptor-binding efficacy. Such genetic variations are of growing interest in periodontal research, as they may influence individual susceptibility to periodontitis and modulate disease severity through altered immune regulation [19]. This genetic perspective not only enhances understanding of disease pathophysiology but also holds potential for identifying novel diagnostic biomarkers and therapeutic targets.

The relevance of *IL-35* extends beyond periodontology. It plays a critical role in shaping immune responses in various immunological contexts, including inflammatory, autoimmune, infectious, septic, and neoplastic diseases [25]. Cytokines are central to immune system communication, mediating the intercellular signaling that governs both innate and adaptive immune responses. Their capacity to maintain immune homeostasis or drive immunopathology depends on the balance between pro-inflammatory and anti-inflammatory mediators [26].

In this regulatory network, *IL-35* acts in concert with other immunosuppressive cytokines such as *IL-10* and transforming growth factor-beta (*TGF-β*). Unlike other IL-12 family members, which predominantly enhance inflammatory responses and facilitate T helper 1 (Th1) or Th17 differentiation, *IL-35* uniquely suppresses T-cell proliferation and pro-inflammatory cytokine production, contributing to immune tolerance [27]. *IL-35*, composed of the p35 and *EBI3* subunits, exemplifies this structural diversity, with its unique configuration contributing to its distinct immunosuppressive function [28].

Experimental evidence from murine models has further underscored *IL-35*’s critical role in immune regulation. Mice deficient in either IL-12p35 or *EBI3* exhibit significantly impaired Treg function and reduced immunosuppressive capacity, leading to increased susceptibility to chronic inflammation [28]. These findings confirm that *IL-35* is not merely a passive anti-inflammatory molecule but an active and essential element of the immune regulatory network.

Given its broad immunosuppressive properties and involvement in diverse pathological states, *IL-35* continues to be a focal point of investigation in immunology, oncology, and infectious disease. In the context of periodontal disease, ongoing research into the functional and genetic regulation of *IL-35* may yield critical insights into individual variability in disease susceptibility, the dynamics of periodontal inflammation, and the development of targeted therapeutic strategies. *IL-35* plays a pivotal role in the regulation of immune responses across a broad spectrum of chronic, autoimmune, and inflammatory diseases. As a uniquely immunosuppressive member of the IL-12 cytokine family, *IL-35* contributes to the maintenance of immune homeostasis by inhibiting pro-inflammatory T-cell subsets, particularly Th17 cells, and by promoting the expansion of regulatory immune cells. Its ability to suppress inflammatory cytokine production and foster immune tolerance underscores its relevance in limiting tissue damage and disease progression [25,27,29]. Given its involvement in modulating host responses in conditions such as periodontitis, rheumatoid arthritis, inflammatory bowel disease, and certain cancers, *IL-35* represents a promising target for diagnostic, prognostic, and therapeutic applications aimed at restoring immunological balance in chronic inflammatory states. In Table 1 the main characteristics of Interleukin 35 are demonstrated.

## 4. *IL-35* in Systemic Immune-Mediated and Neoplastic Diseases

Emerging research underscores *interleukin-35* (*IL-35*) as a cytokine of substantial immunomodulatory potential, with implications for the progression, severity, and prognosis of a diverse range of pathological conditions. Through its dual functions—suppressing pro-inflammatory pathways and enhancing regulatory immune responses—*IL-35* operates at the nexus of inflammatory, autoimmune, neoplastic, and infectious diseases [28,29]. The following is an overview of *IL-35*’s involvement in selected disease states, as documented in the current literature:

- Breast Cancer:

Elevated serum *IL-35* levels have been linked to adverse clinical outcomes in breast cancer patients. Increased *IL-35* concentrations correlate with accelerated tumor progression and reduced survival rates, suggesting a role in facilitating tumor immune escape [30].

- Pancreatic Cancer:

In pancreatic cancer, higher *IL-35* levels are significantly associated with metastatic disease [31]. *IL-35*^+^ regulatory B cells (Bregs) have been implicated in shaping the immunosuppressive tumor microenvironment, thereby contributing to disease pathogenesis [32].

- Lung Cancer:

In non-small-cell lung cancer (NSCLC), overexpression of the *EBI3* subunit is associated with poorer prognosis, identifying it as a potential prognostic marker [33]. Additionally, elevated serum *IL-35* levels are positively correlated with disease progression in NSCLC, reinforcing its role in tumor-associated immunoregulation [34].

- Rheumatoid Arthritis (RA):

Patients with RA typically exhibit reduced circulating *IL-35* levels [35,36]. A negative correlation between *IL-35* concentrations and disease activity has been reported, suggesting a protective and regulatory function for *IL-35* in mitigating inflammatory responses [35,37].

- Systemic Lupus Erythematosus (SLE):

In individuals with SLE, lower serum *IL-35* levels and diminished frequencies of *IL-35*-producing Bregs are associated with more severe disease manifestations [38]. Furthermore, a specific single-nucleotide polymorphism (SNP) in the *EBI3* gene (rs4740) has been linked to increased risk of renal and hematologic involvement, highlighting a genetic contribution to *IL-35* function in SLE [39].

- Type 1 Diabetes Mellitus:

Markedly decreased serum *IL-35* concentrations in patients with type 1 diabetes point to impaired anti-inflammatory regulation, potentially facilitating autoimmune-mediated destruction of pancreatic β-cells [40].

- Atherosclerosis:

Individuals with advanced atherosclerosis show reduced *IL-35* levels, which may hinder the resolution of vascular inflammation and promote instability of atherosclerotic plaques [41].

- Hepatitis B and C:

In hepatitis B, elevated levels of *IL-35*-producing CD4^+^ T cells and B cells have been observed, implicating the cytokine in immune modulation and viral persistence [42,43]. *IL-35* expression has also been linked to chronic viral carriage [44]. In hepatitis C virus (HCV) infection, *IL-35* appears to reduce pro-inflammatory cytokine production, contributing to the attenuation of hepatic inflammation [45].

- Chronic Obstructive Pulmonary Disease (COPD):

Serum *IL-35* levels, alongside IL-17 and *IL-10*, exhibit significant correlations with the clinical features of COPD. These cytokines are currently being explored as potential biomarkers for disease monitoring and therapeutic response [46].

**Figure 2:**
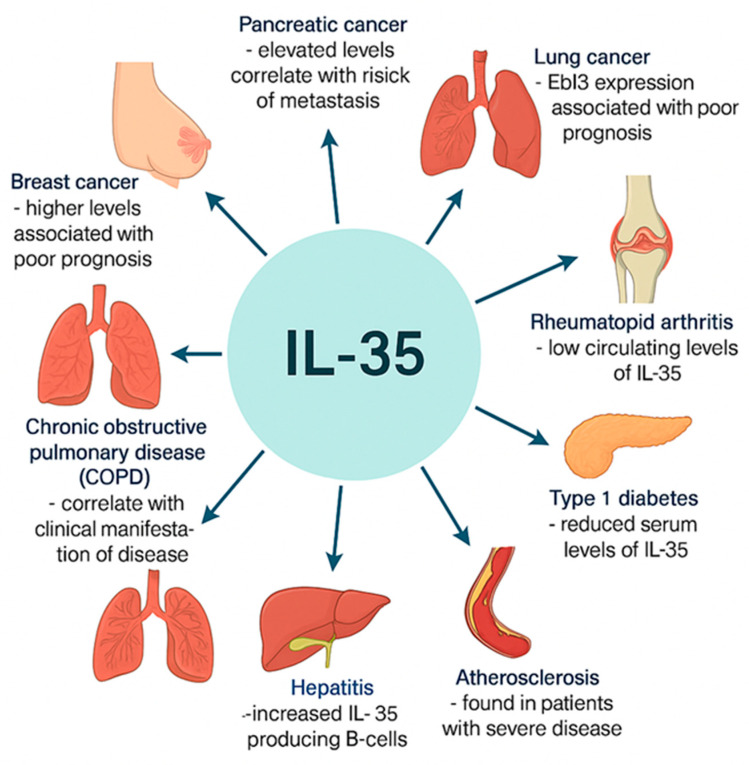
Immunomodulatory role of *IL-35* across major human diseases.

Taken together, these findings highlight the pleiotropic nature of *IL-35* in immune regulation. In autoimmune and pro-inflammatory disorders, *IL-35* predominantly exerts protective, anti-inflammatory effects that mitigate tissue damage and disease severity (Figure 2). Conversely, in malignancies, elevated *IL-35* expression may contribute to tumor progression by fostering an immunosuppressive microenvironment that enables immune evasion. These context-dependent functions position *IL-35* as both a compelling target for therapeutic intervention and a promising biomarker in the evolving field of personalized medicine.

## 5. *IL-35* Gene Polymorphisms and Their Relevance in Immune-Mediated Diseases and Periodontitis

Polymorphisms within the genes encoding *interleukin-35* (*IL-35*) have garnered increasing scientific interest due to their potential roles in modulating individual susceptibility to a range of immune-mediated and inflammatory diseases. *IL-35*, a heterodimeric cytokine composed of the *EBI3* and *IL-12A* (p35) subunits, is recognized for its potent anti-inflammatory and immunosuppressive properties [47]. Genetic variants affecting either subunit may alter cytokine expression or function, thereby influencing immune regulation and disease progression. A notable example of this genetic influence is seen in a study investigating *IL-35* gene polymorphisms in relation to coronary artery disease (CAD) within a Mexican population. The study focused on polymorphisms in the *IL-12A* gene (rs2243115, rs568408, rs2243123, and rs583911) and the *EBI3* gene (rs428253, rs4740, and rs4905). Several of these polymorphisms were significantly associated with a reduced risk of developing CAD, suggesting a protective role in cardio-vascular inflammation. Moreover, some allelic variants were correlated with altered serum *IL-35* levels among healthy individuals, highlighting their potential functional relevance in regulating cytokine production [47].

Further insights into *IL-35*’s genetic impact have emerged from studies on uveitis, particularly Vogt–Koyanagi–Harada (VKH) syndrome—an autoimmune condition targeting melanocyte-containing tissues. Genetic analysis revealed a significant association between the rs4740 polymorphism in the *EBI3* gene and susceptibility to VKH syndrome, reinforcing the hypothesis that genetic variations affecting *IL-35* may impair immune tolerance and contribute to ocular autoimmunity [48].

In contrast, current knowledge concerning the role of *IL-35* gene polymorphisms in periodontal disease remains limited. While some studies have examined associations between single-nucleotide polymorphisms (SNPs) in *EBI3* or *IL-12A* and periodontitis susceptibility, the data are preliminary. Investigations have primarily focused on *IL-35* expression in patients with varying severities and progression rates of periodontitis. However, due to small cohort sizes (e.g., n = 60), the statistical power of these studies remains limited, and authors have consistently emphasized the need for larger, multi-center trials involving genetically diverse populations to establish definitive associations [49]. Despite the increasing attention *IL-35* has received in recent years, human-based studies investigating its association with autoimmune diseases remain limited. However, experimental models in rodents provide important insights into its immunoregulatory potential. In particular, *IL-35* appears to mediate anti-inflammatory effects through interleukin-10 (*IL-10*)-dependent mechanisms, suggesting a shared regulatory axis that could play a protective role in immune-mediated diseases. Despite these constraints, *IL-35* continues to attract attention in periodontal biomarker research. In a study by Yuvashri et al., salivary *IL-35* levels were found to be significantly elevated in both smokers and non-smokers with periodontitis [6]. This consistent increase across subgroups supports the potential utility of *IL-35* as a non-invasive biomarker for disease diagnosis and monitoring [49]. While further validation is necessary, these findings contribute to the growing body of literature positioning *IL-35* as both a candidate biomarker and a genetic target in the translational research landscape of periodontology. In addition to its diagnostic potential, *IL-35* expression is regulated by immune stimuli such as interferon-gamma (IFN-γ) and the activation of Toll-like receptors 3 and 4 (TLR3 and TLR4), highlighting its responsiveness to inflammatory cues [29,50]. Furthermore, its immunosuppressive properties—also observed in contexts like tumor immunity where *IL-35* limits T-cell proliferation and effector function—underscore its broader relevance in immune homeostasis and potential applications in periodontal immuno-pathology [51].

## 6. *IL-35* Expression and Genetic Polymorphisms in Periodontitis

In the context of periodontitis, *interleukin-35* (*IL-35*) has been shown to exert a significant anti-inflammatory effect. Shindo et al. demonstrated that *IL-35* contributes to the immunomodulation of periodontal disease by inhibiting the expression of pro-inflammatory cytokines within periodontal tissues [52]. Complementary findings from Cafferata, using a murine model, revealed that *IL-35* plays a role in suppressing alveolar bone resorption—one of the principal pathological features of advanced periodontitis [53].

Since its discovery, *IL-35* has attracted growing attention for its role in various disease processes and the cell populations involved in its production. The cytokine has been implicated in a broad spectrum of disorders, including acute myeloid leukemia, allergic airway inflammation, colorectal carcinoma, coronary artery disease, lung carcinoma, melanoma, and smoking-related pulmonary inflammation [33,40,51,52,53,54,55,56,57].

In periodontology specifically, *IL-35* has been detected in both gingival tissue and gingival crevicular fluid (GCF) of patients with periodontitis, underscoring its local involvement in oral immune regulation [52]. A study by Kaustubh et al. evaluated *IL-35* mRNA expression in GCF from 15 periodontally healthy individuals, 15 gingivitis patients, and 15 periodontitis patients [58]. Their findings revealed *IL-35* expression across all groups, with significantly higher levels in those with periodontitis—suggesting a potential role in disease progression [58].

Beyond oral health, *IL-35* polymorphisms have been associated with susceptibility to other inflammatory and infectious diseases. For instance, Bassagh et al. examined the rs3761548 polymorphism in *IL-35* genes in patients with Helicobacter pylori-induced peptic ulcer disease [59]. Their study identified the AA genotype and A-allele as being associated with increased disease susceptibility [59]. These findings highlight the broader relevance of *IL-35* polymorphisms in inflammatory conditions beyond the oral cavity, reinforcing the rationale for exploring similar genetic associations in periodontal diseases. In periodontal research, Kalburgi et al. assessed *IL-35* expression in gingival biopsy specimens from 60 participants, categorized as 20 healthy controls, 20 patients with aggressive periodontitis (Stage III, Grade C), and 20 patients with chronic periodontitis—classified according to the 2018 classification of periodontal and peri-implant diseases [49]. Although the study provided valuable preliminary insights, the authors emphasized the need for larger, statistically powered cohort studies to better evaluate the association between *IL-35* and periodontitis [49].

Nevertheless, findings regarding the role of *IL-35* in periodontal disease remain somewhat contradictory. While some studies suggest that *IL-35* may serve as a biomarker of disease severity, others yield inconclusive results [60]. One factor potentially contributing to this variability is genetic influence on *IL-35* expression. Since periodontal disease shares common pathogenetic mechanisms with other inflammatory and autoimmune conditions, *IL-35* polymorphisms may also be associated with the incidence and severity of other significant systemic diseases. For instance, Xie et al. identified *IL-35* polymorphisms associated with altered serum expression in patients with rheumatoid arthritis [61], underscoring the importance of pursuing similar investigations in periodontal populations. Likewise, Posadas-Sánchez et al. reported an association between the rs428253 polymorphism in *EBI3* and a reduced risk of premature coronary artery disease [47], and Guan et al. found that the rs4740 polymorphism was significantly linked to systemic lupus erythematosus in a Han Chinese cohort [39]. These findings suggest that *IL-35* gene variants may modulate disease susceptibility, providing a rationale for exploring their role in periodontitis. The role of *interleukin-35* (*IL-35*) in periodontitis remains complex, with current data yielding diverse and sometimes contradictory results. Periodontitis, a highly prevalent chronic inflammatory disease, results from intricate interactions between pathogenic oral microbiota and the host immune response. Within periodontitis lesions, plasma cells are the most abundant immune cell subset, comprising approximately 50% of the inflammatory infiltrate. Although plasma cells have traditionally been regarded primarily as antibody producers, emerging evidence highlights their additional capacity to secrete immunoregulatory cytokines [6,16].

A study by Jing et al. provides compelling immunohistochemical and immuno-fluorescent evidence that plasma cells in CP tissues produce the anti-inflammatory cytokines *IL-35* and *IL-37* [62]. Two novel plasma cell subsets were identified: *IL-37*-producing plasma cells (*PIL-37*) and *IL-35*/*IL-37*-co-producing plasma cells (P*IL-35*/*IL-37*), both characterized by the CD138^+^ CD38^+^ IgG^+^ phenotype. Recombinant *IL-35* and *IL-37* demonstrated strong, dose-dependent inhibitory effects on osteoclast formation, suggesting that these cytokines may exert a critical protective role against alveolar bone resorption. These findings reveal a previously underrecognized immunoregulatory function of plasma cells in the periodontal microenvironment and propose *IL-35* and *IL-37* as potential therapeutic targets for halting disease progression and preserving periodontal tissue [62].

In a complementary study, Han et al. examined the immunomodulatory role of CD25^+^ regulatory B cells (Bregs) in the context of periodontitis, focusing on their interaction with Toll-like receptors (TLRs) and their effects on cytokine profiles and T-cell differentiation [63]. Using flow cytometry, ELISA, real-time PCR, and adoptive transfer models, the researchers demonstrated that periodontitis induces a significant expansion of CD25^+^ B-cell subpopulations, which in turn show enhanced production of *IL-10*, *IL-35*, and *TGF-β*. Stimulation through TLR4 and TLR9 further amplified both the differentiation and cytokine secretion capacity of these cells [63]. Notably, adoptive transfer of CD25^+^ B cells into murine models of periodontitis significantly reduced alveolar bone resorption, suppressed local levels of IFN-γ and IL-17, and restored the Th1/Th17/Treg balance within inflamed periodontal tissues. These effects were localized, with no systemic immunological changes observed. Collectively, the findings suggest that CD25^+^ B cells exert a protective and regulatory function in periodontal inflammation via *IL-35*-mediated suppression of pathogenic T-cell responses, identifying TLR signaling and *IL-35* as potential therapeutic targets [63].

Further exploring the immunological relevance of *IL-35* in periodontitis, Hassan et al. investigated the expression of *IL-35* and *IL-39* in both systemically healthy and diabetic individuals with periodontitis to evaluate their potential as biomarkers [64]. The study included 38 patients with Stage III periodontitis and 19 periodontally healthy controls. The periodontitis group was subdivided into two equal cohorts: Group I consisted of patients with Grade C periodontitis and type 2 diabetes mellitus (T2DM), and Group II comprised systemically healthy patients with Grade B periodontitis. *IL-35* and *IL-39* levels were measured in gingival crevicular fluid (GCF) pre- and post-operatively using ELISA [64]. The results indicated that *IL-39* levels were significantly elevated in diabetic patients with periodontitis, suggesting a potential pro-inflammatory role. These levels declined markedly following non-surgical periodontal therapy. In contrast, *IL-35* levels were highest in the healthy control group and lowest in diabetic periodontitis patients, though they increased significantly after treatment. These findings support the hypothesis that *IL-35* plays a protective anti-inflammatory role, while *IL-39* may serve as a marker of inflammatory burden. Both interleukins demonstrated clinical responsiveness to therapy, suggesting their utility as biomarkers for disease activity and treatment outcomes, irrespective of systemic comorbidity [64].

In a related genetic study, Durga et al. assessed the potential association between *IL-35* gene polymorphisms—specifically rs1580809257 and rs1580801731—and susceptibility to periodontitis, with and without concomitant type 2 diabetes mellitus (T2DM) [65]. Ninety-six participants were categorized into three groups: healthy controls, systemically healthy individuals with generalized chronic periodontitis, and patients with periodontitis and T2DM. Genomic DNA was isolated from peripheral blood, and genotyping was conducted using ARMS-PCR and restriction fragment length polymorphism (RFLP) techniques [65]. Genotype and allele frequencies were evaluated using the Chi-square test, and the Hardy–Weinberg equilibrium was confirmed. The analysis revealed no significant differences in genotype or allele frequencies among the groups, suggesting that the selected *IL-35* polymorphisms were not associated with an increased risk of periodontitis, either independently or in the presence of diabetes [65]. Although these findings did not demonstrate a functional role for the tested polymorphisms in *IL-35*-mediated immune modulation, they underscore the importance of expanding genetic association studies to include broader panels of variants and more diverse populations. Such efforts are essential for fully elucidating the genetic contributions to periodontal disease susceptibility and its interaction with systemic conditions such as T2DM [65].

An important contribution to the understanding of *IL-35*’s systemic role in inflammatory disease was made by Maboudi et al., whose cross-sectional study provided valuable negative evidence—an often underappreciated yet essential aspect of genetic and immunological research [66]. The study assessed the systemic immunological profiles of interleukin-23 (IL-23) and *interleukin-35* (*IL-35*) in four participant groups: healthy controls, individuals with type 2 diabetes mellitus (T2DM) alone, individuals with periodontitis alone, and those with both T2DM and periodontitis [66]. The total sample included 72 participants, equally distributed among the groups. Comprehensive periodontal examinations were conducted alongside metabolic and inflammatory assessments, including fasting blood sugar (FBS), hemoglobin A1c (HbA1c), erythrocyte sedimentation rate (ESR), C-reactive protein (CRP), and serum levels of IL-23 and *IL-35*, measured using the enzyme-linked immunosorbent assay (ELISA). The findings indicated no statistically significant differences in serum IL-23 or *IL-35* concentrations between the groups (*p* > 0.05), suggesting that the presence of diabetes, periodontitis, or their combination does not independently or synergistically influence the systemic levels of these cytokines. However, within-group analyses revealed several meaningful correlations: IL-23 was positively associated with clinical attachment loss (CAL) in healthy individuals (r = 0.548, *p* = 0.019); *IL-35* showed a negative correlation with the plaque index in the T2DM-only group (r = −0.578, *p* = 0.012); and IL-23 was inversely correlated with ESR and CRP in the periodontitis subgroups (r ≈ −0.49, *p* < 0.05) [66]. Although systemic *IL-35* levels did not differ significantly by disease status, these findings suggest that *IL-35* may exert localized or context-dependent regulatory effects not reflected in systemic circulation. The study underscores the complexity of cytokine behavior across systemic and local compartments and cautions against relying solely on serum *IL-35* levels as diagnostic markers for periodontitis or diabetes. Further research exploring tissue-level cytokine expression and longitudinal immunological dynamics is warranted to clarify *IL-35*’s specific role in the immunopathogenesis of chronic inflammatory diseases [66].

Taskaldiran et al. conducted a study to evaluate the influence of smoking on the levels of IL-17 and *IL-35*, representing pro-inflammatory and anti-inflammatory cytokines, respectively, in both the saliva and gingival crevicular fluid (GCF) of individuals with periodontitis [67]. The study cohort comprised 19 smokers and 20 non-smokers with periodontitis, alongside 18 periodontally healthy controls. Clinical periodontal parameters were recorded, and cytokine levels were quantified via immunoassay techniques. While salivary levels of IL-17 and *IL-35* did not differ significantly across the groups, distinct patterns emerged in GCF cytokine concentrations. Specifically, GCF levels of both IL-17 and *IL-35* were significantly lower in non-smokers with periodontitis, compared to their smoking counterparts. However, total IL-17 levels in GCF were markedly elevated in both smoker and non-smoker periodontitis groups relative to controls, reflecting an overall heightened inflammatory burden [67]. Intriguingly, GCF *IL-35* levels were significantly higher in non-smokers with periodontitis than in both smokers and healthy controls, suggesting a compensatory anti-inflammatory response in the absence of tobacco exposure. Moreover, strong positive correlations were observed between IL-17 and *IL-35* levels in both saliva and GCF (r = 0.854–0.973, *p* < 0.01), indicating a coordinated interplay between these cytokines within the local inflammatory milieu. These findings suggest that *IL-35*, in conjunction with IL-17, may play a role in modulating immune responses in periodontitis [67]. Notably, the data implies that smoking may suppress local *IL-35* production, thereby exacerbating inflammation and potentially worsening disease severity. The study supports the potential of *IL-35* as a biomarker of periodontal inflammation and tissue homeostasis, while also identifying tobacco exposure as a critical modulator of cytokine expression in periodontal disease. Further studies are warranted to delineate the molecular mechanisms through which smoking alters *IL-35* function in periodontal tissues [67].

In a complementary investigation, Jin et al. examined the expression profile and regulatory role of *IL-35* in both peripheral and local immune compartments of patients with periodontitis, with a particular emphasis on T-cell–mediated immunity, including regulatory T cells (Tregs) and T helper 17 (Th17) cells [68]. The study collected samples of peripheral blood mononuclear cells (PBMCs), gingival tissue, GCF, and serum from CP patients, which were compared to samples from periodontally healthy individuals undergoing extraction of impacted teeth. Using RT-qPCR and ELISA, the researchers observed significantly elevated mRNA expression of *IL-35* subunits in both periodontal tissues and PBMCs of CP patients (*p* < 0.05). In addition, IL-35 protein levels were markedly increased in GCF and serum samples from the periodontitis group (*p* < 0.001). Notably, *IL-35* concentrations were inversely correlated with clinical indicators of disease severity, such as probing depth and clinical attachment loss, suggesting a protective, anti-inflammatory role for *IL-35* in the periodontal environment [68]. These findings support the hypothesis that *IL-35* contributes to immune homeostasis in periodontitis by mitigating excessive inflammatory responses and modulating the balance between pathogenic and regulatory T-cell subsets. The study positions *IL-35* as a promising immunoregulatory mediator and highlights its potential as a therapeutic target in the management of chronic periodontitis. Further research is needed to explore its role in immunomodulatory treatment strategies aimed at restoring periodontal tissue integrity through cytokine regulation [68].

The study by Eriksson et al. investigated the complex interplay between the oral microbiome and host inflammatory mediator profiles in patients diagnosed with chronic periodontitis, with or without concomitant rheumatoid arthritis (RA) [69]. Salivary samples were obtained from 53 patients with both periodontitis and RA and 48 patients with periodontitis alone. The samples were analyzed using 16S rRNA gene sequencing to characterize microbial communities and multiplex bead-based immunoassays to quantify cytokine expression. To identify distinct patterns, the authors employed a combination of statistical and machine learning approaches, including Principal Coordinate Analysis (PCoA), DESeq2, orthogonal partial least squares discriminant analysis (OPLS-DA), and sparse PLS-DA (sPLS-DA) [69]. These analyses revealed significant differences in the microbial profiles between the two groups. Patients with periodontitis alone exhibited enrichment of Alloprevotella, Prevotella, Haemophilus, and Actinomyces, while those with RA and periodontitis showed increased relative abundance of Granulicatella, Veillonella, Megasphaera, Fusobacterium nucleatum, and amplicon sequence variants (ASVs) from rarer genera such as Sphingomonas, Novosphingobium, and Aquabacterium. Importantly, the study found that several inflammatory mediators—including *interleukin-35* (*IL-35*), *TWEAK*/*TNFSF12*, *interferon-α2* (IFN-α2), *pentraxin-3*, *gp130*/*sIL-6Rb*, *interleukin-19* (*IL-19*), and soluble TNF receptor-1 (sTNF-R1)—were significantly elevated in patients with both RA and periodontitis compared to those with periodontitis alone. Moreover, strong correlations were identified between specific bacterial ASVs and inflammatory mediator levels, suggesting the presence of distinct microbial inflammation signatures in the context of comorbid disease [69]. These findings indicate that *IL-35* may participate in the systemic amplification of inflammation observed in patients with coexisting RA and periodontitis, potentially functioning as a compensatory immunoregulatory cytokine. The study further underscores the value of integrated analysis of microbial and cytokine data in providing a more comprehensive and accurate biomarker profile for complex inflammatory conditions. This integrative, system-level approach holds significant promise for the development of personalized diagnostic tools and therapeutic strategies that address both local and systemic aspects of chronic inflammatory disease [69].

Mitani et al. focused on the local expression of *interleukin-35* (*IL-35*) in gingival crevicular fluid (GCF) and gingival tissues of patients with periodontitis, aiming to evaluate its role in cytokine-mediated immune regulation [24]. Using ELISA for protein quantification and qPCR for gene expression analysis, the study specifically measured levels of *IL-35* subunits—*EBI3* and *IL12A*—in both inflamed and healthy periodontal tissues. The results demonstrated that *IL-35* levels in GCF were significantly elevated in patients with periodontitis compared to healthy controls (*p* < 0.01). In parallel, the mRNA expression of *IL-35* subunits (*EBI3* and *IL12A*) was markedly upregulated in diseased gingival tissues, indicating active local production of *IL-35* at inflamed sites. Notably, *IL-35* levels positively correlated with clinical indicators of disease severity, including probing depth and clinical attachment level (CAL). This correlation suggests that *IL-35* may be produced in response to the increasing inflammatory burden, possibly functioning as a compensatory anti-inflammatory mechanism. These findings reinforce the hypothesis that *IL-35* is actively involved in the local immune response to periodontitis, likely serving to counterbalance pro-inflammatory mediators such as IL-17. Unlike IL-27, which was undetectable in this context, *IL-35* appears to be functionally relevant in the periodontal microenvironment, highlighting its potential utility as a biomarker of disease activity and a target for therapeutic modulation [24].

In the study by Ho et al., *IL-35* levels were assessed in both gingival crevicular fluid (GCF) and plasma among individuals with healthy periodontium, gingivitis, and periodontitis [70]. While the study did not report significant differences in *IL-35* concentrations within GCF across the groups, a noteworthy finding was the progressive increase in plasma *IL-35* levels with advancing periodontal disease severity. This observation suggests that *IL-35* may contribute to systemic immune regulation in response to local periodontal inflammation. The authors propose that *IL-35* may function as a circulating anti-inflammatory cytokine, mobilized to mitigate systemic effects of chronic oral inflammation, thus supporting its potential role as a biomarker for systemic immune activity in the context of periodontitis [70].

In a separate cross-sectional analysis, Altaca et al. evaluated *IL-35* levels in GCF alongside IL-6 and IL-17 in a cohort of 60 patients with Stage III and IV periodontitis and 30 periodontally healthy controls [71]. Clinical parameters—including probing depth (PD) and clinical attachment loss (CAL)—were significantly elevated in the periodontitis group, confirming disease severity. The study revealed that *IL-35* levels in GCF were significantly elevated in periodontitis patients compared to controls (*p* < 0.001). Moreover, logistic regression analysis demonstrated a statistically significant association between elevated *IL-35* levels and the presence of periodontitis (odds ratio = 1.261; 95% CI = 1.110–1.434; *p* < 0.001). Interestingly, while IL-17 levels correlated directly with clinical measures of disease severity (e.g., pocket depth), *IL-35* did not show significant correlation with individual clinical indices. Nonetheless, its consistently elevated levels in patients with moderate to severe periodontitis point to a possible compensatory anti-inflammatory response to chronic inflammation. These results highlight *IL-35*’s potential as a local biomarker for the presence of advanced periodontal disease, even if not directly tied to the extent of tissue destruction. Its regulatory profile may reflect broader immune adaptation rather than localized disease severity [71].

Kamiya et al. investigated the role of *interleukin-35* (*IL-35*) in osteoclastogenesis, a critical process underlying alveolar bone resorption in chronic inflammatory diseases such as periodontitis and rheumatoid arthritis (RA) [72]. Although *IL-35* is widely recognized for its immunosuppressive function, primarily via regulatory T cells, its direct effects on osteoclast differentiation had not been fully elucidated prior to this study. Using the RAW264.7 murine macrophage cell line, the researchers evaluated the effects of *IL-35* in the presence of receptor activator of NF-κB ligand (RANKL), a key stimulator of osteoclast formation. A combination of tartrate-resistant acid phosphatase (TRAP) staining, hydroxyapatite resorption assays, and qPCR-based gene expression analysis was used to quantify osteoclast activity, while Western blotting was employed to assess intracellular signaling pathways [72]. The results revealed that co-stimulation with *IL-35* and RANKL significantly enhanced osteoclastogenesis compared to RANKL alone. This effect was mechanistically linked to increased phosphorylation of extracellular signal-regulated kinase (ERK) and p38 mitogen-activated protein kinase (MAPK), two signaling pathways known to promote osteoclast differentiation. Importantly, enhanced osteoclastogenic activity was attenuated by ERK inhibition, confirming that *IL-35* contributes directly to osteoclastogenesis via MAPK signaling [72]. These findings are particularly noteworthy because they contrast with previous studies reporting *IL-35*’s inhibitory effects on bone resorption, suggesting a context-dependent duality in its function. Kamiya et al. propose that *IL-35* may exert either protective or pathogenic effects, depending on the local cytokine environment and cellular interactions [72]. In the context of periodontitis, this implies that *IL-35* could participate in both immune regulation and bone destruction, depending on disease stage and tissue microenvironment. This study highlights the complexity of *IL-35*’s immunobiological role and underscores the need for further investigation to define the conditions under which it shifts from an anti-inflammatory mediator to a promoter of tissue degradation. Such insights will be essential for determining whether *IL-35* can be safely and effectively targeted in periodontal therapy [72].

The study by Köseoğlu et al. offers valuable insights into the local and systemic distribution of *interleukin-35* (*IL-35*) across varying stages of periodontal health [73]. Specifically, the research focused on the differential behavior of *IL-35* in gingival crevicular fluid (GCF), saliva, and plasma among systemically healthy, non-smoking individuals. Participants were stratified into three clinical groups—periodontally healthy, gingivitis, and periodontitis—with 20 individuals per group. Clinical periodontal parameters were assessed, and *IL-35* concentrations in biological fluids were measured using ELISA. The findings revealed a complex compartmental pattern of *IL-35* expression. While the total amount of *IL-35* in GCF was highest in the periodontitis group (*p* = 0.04), the concentration of *IL-35* in GCF—reflecting actual local levels after accounting for volume—was significantly higher in the healthy group (*p* = 0.002) [73]. This suggests that although *IL-35* production is increased in disease, its effectiveness may be diminished due to dilution or impaired regulatory function in the inflamed environment. Similarly, salivary *IL-35* levels were highest in periodontally healthy individuals and lowest in those with periodontitis (*p* < 0.001), reinforcing the hypothesis that *IL-35* expression is downregulated during active inflammation or insufficient to counterbalance pro-inflammatory mediators. In contrast, plasma *IL-35* levels did not differ significantly among groups (*p* > 0.05), suggesting that systemic *IL-35* may not reflect local periodontal immune status. Importantly, positive correlations were observed between the total *IL-35* in GCF and clinical parameters such as probing depth (PD) (r = 0.338, *p* = 0.03) and plaque index (PI) (r = 0.374, *p* = 0.005), indicating a potential role for *IL-35* in modulating local inflammatory burden [73]. These findings support the notion that *IL-35* contributes to the local regulation of periodontal inflammation, with elevated total levels during disease likely reflecting an attempted anti-inflammatory response, while higher concentrations in health may signify a protective immunoregulatory baseline. The study underscores the importance of evaluating cytokine dynamics across multiple biological compartments and highlights *IL-35*’s potential utility as a local biomarker for disease activity and resolution in periodontitis [73].

The study by Cafferata et al. offers compelling in vivo evidence for the protective effects of *interleukin-35* (*IL-35*) in the context of periodontitis, specifically through its ability to modulate T-cell–mediated immune responses and prevent alveolar bone resorption [53]. Utilizing a murine model of ligature-induced periodontitis, the researchers investigated the therapeutic potential of *IL-35* by administering the cytokine either locally or systemically, comparing outcomes against both untreated periodontitis-affected mice and non-ligated healthy controls. Alveolar bone loss was quantitatively assessed using micro-computed tomography (micro-CT) and scanning electron microscopy, while qPCR, ELISA, and flow cytometry were employed to evaluate local immune cell populations and cytokine profiles [53]. The results revealed that *IL-35* treatment significantly inhibited alveolar bone resorption. Mechanistically, this effect was associated with a reduction in pro-inflammatory Th17 cells and their associated cytokines, alongside a concurrent increase in regulatory T cells (Tregs) and anti-inflammatory mediators within periodontal tissues. These findings strongly suggest that *IL-35* exerts its protective effects by rebalancing the Th17/Treg axis, shifting the immune environment from a pro-inflammatory, tissue-destructive phenotype to one characterized by immune regulation and tissue preservation [53]. The study highlights *IL-35*’s potential as a therapeutic agent for managing periodontitis, particularly in cases where disease progression is driven by dysregulation of T-cell subsets. This work contributes to a growing body of evidence supporting *IL-35*’s role as a targetable immunomodulatory cytokine in chronic inflammatory conditions affecting the periodontium [53]. Table 2 presents insights into major studies related to Interleukin 35 and its relation to periodontitis.

## 7. *IL-35* Response to Periodontal Therapy: A Marker of Resolution or Modulator of Healing

As an immunoregulatory cytokine, *interleukin-35* (*IL-35*) has gained considerable attention not only for its role in the pathogenesis of periodontitis but also for its behavior in response to periodontal therapy. Monitoring changes in *IL-35* levels following therapeutic intervention offers valuable insight into its potential as a biomarker of treatment efficacy and as a possible modulator of post-treatment immune resolution [74]. A growing body of research suggests that *IL-35* levels may fluctuate in accordance with inflammatory status—declining as inflammation subsides and immune homeostasis is restored.

In a comparative clinical study, Raj et al. evaluated *IL-35* levels in gingival crevicular fluid (GCF) and serum across individuals with varying periodontal conditions, and assessed changes following non-surgical periodontal therapy (NSPT) [74]. Sixty participants were divided into three groups: periodontally healthy, gingivitis, and chronic periodontitis. *IL-35* concentrations were measured at baseline in all groups, and again six weeks post-therapy in the periodontitis group, using enzyme-linked immunosorbent assay (ELISA) [74]. Baseline *IL-35* levels were highest in untreated periodontitis patients, suggesting a link between elevated *IL-35* and active inflammation. Notably, post-NSPT *IL-35* levels in the periodontitis group declined significantly, approaching those of the healthy and gingivitis groups. This reduction indicates that *IL-35* is responsive to periodontal inflammatory changes and may reflect disease resolution. The findings support *IL-35* in GCF as a potential non-invasive biomarker for disease severity and therapeutic response. Additionally, its modulation post therapy suggests a role in immune rebalancing during healing. Collectively, these results position *IL-35* as a promising candidate for diagnostic and prognostic use in personalized periodontitis management [74].

In a prospective clinical study, Goswamy et al. evaluated temporal changes in *interleukin-35* (*IL-35*) levels within gingival crevicular fluid (GCF) following non-surgical periodontal therapy (NSPT) in patients with generalized periodontitis [75]. Twenty participants with moderate to severe disease underwent full-mouth NSPT. GCF samples were collected from the deepest periodontal pockets at baseline, and at one, two, and three weeks post treatment. Over this period, significant clinical improvements were observed in plaque index, gingival index, probing depth, and clinical attachment loss, reflecting inflammation resolution. Concurrently, *IL-35* levels in GCF increased progressively and significantly, with a highly significant post-therapy elevation (*p* < 0.001) [75]. Notably, *IL-35* levels were inversely correlated with clinical markers of disease severity, suggesting higher *IL-35* levels were linked to improved periodontal outcomes. These results indicate *IL-35* is upregulated during healing and supports its role as an anti-inflammatory cytokine involved in immune restoration. The post-treatment rise highlights its potential as a biomarker for monitoring therapeutic efficacy and tissue recovery, supporting *IL-35*’s dual role as a diagnostic and prognostic indicator in periodontal care [75].

Durgapal et al. investigated salivary *IL-35* levels in relation to periodontal status and response to NSPT [76]. Seventy participants were divided into three groups: periodontally healthy controls, gingivitis patients, and those with Stage II periodontitis. Saliva samples were analyzed using ELISA, with follow-up sampling at 12 weeks post-therapy in the periodontitis group. At baseline, *IL-35* levels were significantly higher in healthy individuals than in those with gingivitis or periodontitis (*p* < 0.05), suggesting a protective role in maintaining immune equilibrium. Conversely, lower *IL-35* levels were observed in individuals with periodontal disease, consistent with inflammation. Following NSPT, the periodontitis group showed a significant increase in *IL-35*, reaching 29.47 ± 17.88 pg/mL, indicating a shift toward anti-inflammatory regulation [76]. These findings further support *IL-35*’s potential as a salivary biomarker for periodontal health and treatment response.

Similarly, a randomized controlled clinical trial by Jadhav et al. evaluated GCF *IL-35* levels among periodontally healthy individuals, gingivitis patients, and patients with Stage III periodontitis, both before and after NSPT [77]. A total of 60 participants were equally distributed among the three groups. *IL-35* concentrations and clinical parameters were assessed via ELISA and periodontal examination, respectively. At baseline, *IL-35* levels were lowest in the periodontitis group, intermediate in gingivitis patients, and highest in healthy controls, illustrating a negative correlation between *IL-35* and disease severity. Post-treatment analysis revealed significant clinical improvement and a notable increase in *IL-35* levels in both the gingivitis and periodontitis groups [77]. The results of both studies collectively reinforce the hypothesis that *IL-35* plays a protective, anti-inflammatory role in periodontal homeostasis and may serve as a robust biomarker for disease activity, immune regulation, and therapeutic efficacy. The elevation of *IL-35* levels following NSPT provides further evidence of its potential involvement in the resolution of inflammation and stabilization of periodontal tissues.

In their clinical investigation, Thakare et al. evaluated *interleukin-35* (*IL-35*) levels in gingival crevicular fluid (GCF) across individuals with varying degrees of periodontal health, including healthy subjects, gingivitis patients, and those with chronic periodontitis (n = 15 per group) [78]. Clinical parameters—such as probing depth, clinical attachment loss, bleeding indices, and plaque scores—were documented, and *IL-35* concentrations were measured via ELISA. The findings revealed that *IL-35* levels were highest in periodontally healthy individuals, with a progressive decline in gingivitis and chronic periodontitis patients. This trend was consistent not only across groups but also within different sites (healthy vs. inflamed) in the same individuals, suggesting that *IL-35* levels are sensitive to localized inflammatory status. These results support the hypothesis that *IL-35* contributes to the maintenance of periodontal homeostasis, and that its reduced expression is linked to increased gingival inflammation [78]. Therefore, GCF *IL-35* levels may serve as a non-invasive biomarker for identifying periodontal disease activity and distinguishing between health, gingivitis, and active periodontitis.

## 8. Discussion

Periodontitis is a disease with a significant impact on individuals in terms of masticatory function, esthetics, self-esteem, and socializing. Its diagnosis implements clinical and radiographic investigations that often are subject to subjective interpretation or technical error. The conventional diagnostic techniques often do not show the present activity of the disease but only the history of such activity [79]. The complex etiopathogenetic mechanisms of periodontitis doubt the conventional methods as sufficient ones and are aiming to identify precise methods showing susceptibility, activity, and treatment response [80].

The recent statement on the individual’s health relies on the understanding of the personalized approach both in relation to diagnostics and treatment. Personalized medicine is gaining attention by proposing targeted therapies tailored to the personal needs of patients. In terms of suggesting the best therapeutic solution, it uses personal information including not only the history of the disease but also lifestyle and environmental information and genetic data [81,82]. Recent diagnostic methods could be supplemented with the benefits that the biomarkers hold in terms of the detection of individuals suspectable to a risk of rapid tissue destruction. Modern science is oriented toward seeking diagnostic markers that assess the patient’s compliance with treatment, surrogate markers, including inflammatory, tissue response products, etc. [83]. On the other hand, defining predictive markers for patients at risk of developing periodontitis or those at high risk of disease progression could be helpful in disease prevention [84]. Genetic biomarkers can be used as a prognostic marker in treatment planning to enhance the treatment and personalized maintenance intervals [80,85,86].

*IL-35* meets these challenges as a candidate genetic biomarker for disease susceptibility and disease activity and treatment response. *Interleukin-35* (*IL-35*) has emerged as a multifaceted immunoregulatory cytokine with distinct, context-dependent roles across a wide spectrum of pathological conditions. Its dual capacity to suppress pro-inflammatory responses while enhancing regulatory immune functions places *IL-35* at a critical interface between immune tolerance and immune evasion. This dichotomy is particularly evident in the contrasting roles *IL-35* plays in autoimmune/inflammatory diseases versus malignancies [78].

In autoimmune diseases such as rheumatoid arthritis (RA) and systemic lupus erythematosus (SLE), *IL-35* appears to exert a protective role. Reduced serum *IL-35* levels in RA patients are consistently linked to heightened disease activity, indicating a deficiency in immunosuppressive regulation during inflammatory flares [35,36,37]. Similarly, in SLE—a systemic condition marked by immune dysregulation—lower *IL-35* levels and fewer *IL-35*-producing regulatory B cells are associated with more severe disease phenotypes [38]. A variant in the *EBI3* gene (rs4740), which increases susceptibility to renal and hematologic involvement in SLE, further highlights *IL-35*’s functional and heritable relevance in autoimmune regulation [38,87]. *IL-35* is increasingly viewed as a therapeutic target in cancer, inflammation, infections, and autoimmune disorders, including type 1 diabetes mellitus (T1DM). Although its role in T1DM is not yet fully understood, reduced *IL-35* levels suggest weakened regulatory control over autoimmune responses against pancreatic β cells [40]. Similarly, in atherosclerosis, diminished *IL-35* may sustain vascular inflammation and destabilize plaques, contributing to disease progression [41,87,88]. Together, these findings support *IL-35*’s role as a negative regulator of immune activation and chronic tissue damage. In contrast, *IL-35* can promote disease progression in neoplastic conditions. Produced by regulatory T cells (Tregs), it supports tumor immune evasion. In gastric adenocarcinoma (GA), elevated *IL-35* levels correlate with disease progression. A study of the *Foxp3* gene polymorphism (rs3761548) found that the AA and AC genotypes were associated with higher risks of GA and increased levels of *IL-35*, *IL-10*, and *TGF-β*, suggesting that *IL-35*—modulated by Treg-related genetic variants—enhances immunosuppression within tumors [89]. Similarly, in breast and pancreatic cancers, elevated *IL-35* correlates with metastasis and poor survival outcomes [30,31,32], likely due to the influence of *IL-35*-producing regulatory B cells (Bregs) in suppressing anti-tumor responses. In non-small-cell lung cancer (NSCLC), *EBI3* overexpression and elevated circulating *IL-35* levels are linked to advanced disease and poor prognosis [33,34], suggesting *IL-35* reflects tumor burden and may facilitate immune evasion.

A similar immunosuppressive role is seen in chronic viral infections, such as hepatitis B and C. *IL-35*-producing CD4^+^ T cells and B cells are elevated in hepatitis B, potentially contributing to viral persistence and chronicity [42,43,44]. In hepatitis C, *IL-35*-mediated downregulation of pro-inflammatory cytokines may attenuate liver inflammation but also impede effective viral clearance [45]. These roles underscore the delicate balance *IL-35* must maintain between immune tolerance and pathogen eradication. In chronic obstructive pulmonary disease (COPD), *IL-35* levels correlate with disease characteristics alongside IL-17 and *IL-10*, positioning *IL-35* as a potential biomarker for disease monitoring and therapeutic targeting [46]. This suggests that even in non-autoimmune, chronic inflammatory conditions, *IL-35* could serve a modulatory function within complex cytokine networks [46,47]. Taken together, these findings illustrate the pleiotropic and context-specific functions of *IL-35* in immune regulation. In autoimmune and inflammatory conditions, *IL-35* generally acts as a suppressive cytokine that mitigates immune-mediated damage and improves clinical outcomes. In contrast, its upregulation in cancer and chronic infections appears to support immune evasion and disease progression. This functional dualism underscores the necessity for disease-specific strategies when considering *IL-35* as a therapeutic target or biomarker.

In conclusion, *IL-35* holds a significant promise in translational immunology. Its role as a biomarker for disease progression and therapeutic response, combined with its therapeutic potential in modulating immune activity, makes *IL-35* a compelling target for further investigation. However, its biphasic nature—protective in some diseases and pathogenic in others—highlights the need for context-driven therapeutic approaches and reinforces the value of personalized medicine in managing complex immune-related disorders.

*Interleukin-35* (*IL-35*) has emerged as a cytokine of considerable interest in periodontal research, owing to its dual roles in immune suppression and tissue regulation. Its expression patterns, cellular sources, and genetic variations offer valuable insight into its complex role in periodontitis and its potential as both a biomarker and therapeutic target. Numerous studies have demonstrated elevated *IL-35* levels in gingival tissues, gingival crevicular fluid (GCF), and serum of patients with periodontitis, suggesting its involvement in modulating local immune responses. Shindo et al. reported *IL-35*’s ability to suppress pro-inflammatory cytokine expression in periodontal tissues [52], while Cafferata et al. found that *IL-35* administration in a murine model significantly reduced alveolar bone loss, mainly through the modulation of T helper (Th17) and regulatory T-cell (Treg) populations [53]. These findings support *IL-35*’s immunosuppressive and bone-protective functions and its inhibition of periodontal tissue destruction [90].

At the local level, *IL-35* has been shown to be actively produced by plasma cells [62], regulatory B cells (Bregs) [63], and other immune cells within periodontal lesions. The discovery of *IL-35*-producing plasma cell subsets—such as *PIL-37* and P*IL-35*/*IL-37*—adds to the growing understanding of immune cell plasticity in chronic periodontitis. Similarly, Han et al. demonstrated that TLR4 and TLR9 stimulation enhances *IL-35* production by CD25^+^ Bregs, contributing to local immune suppression and T-cell modulation [63]. Several studies indicate that *IL-35* levels are dynamically regulated in response to treatment and disease status. For instance, Hassan et al. observed increased *IL-35* levels following non-surgical periodontal therapy, especially in patients with periodontitis and type 2 diabetes mellitus (T2DM), further reinforcing *IL-35*’s responsiveness to clinical intervention [64]. Contrastingly, Maboudi et al. found no significant differences in systemic *IL-35* levels across groups with or without diabetes and/or periodontitis, suggesting that *IL-35*’s immunoregulatory effects may be largely localized and context-dependent [65,66].

Genetic studies provide additional complexity. Kalburgi et al. and Durga et al. found no significant associations between selected *IL-35* polymorphisms and periodontitis susceptibility in small cohorts [49,65]. However, evidence from systemic diseases suggests a potential genetic component: Bassagh et al. linked rs3761548 polymorphisms to Helicobacter pylori-associated disease [59], and other studies reported associations between *IL-35* variants (e.g., rs428253, rs4740) and autoimmune conditions such as RA and SLE [39,47,61]. These findings indicate that while *IL-35* polymorphisms may not currently serve as strong genetic predictors in periodontitis, broader and more diverse genomic analyses are warranted.

Conflicting results exist regarding *IL-35*’s correlation with disease severity. Some reports found elevated *IL-35* levels in periodontitis, suggesting a compensatory response to chronic inflammation [24,71]. However, others—such as Ho et al. and Köseoğlu et al.—suggest *IL-35* expression is higher in health or early disease stages, possibly indicating a protective baseline function that diminishes as inflammation overwhelms local immune control [70,73]. These contrasting outcomes may reflect differences in sample origin (e.g., saliva vs. GCF vs. serum), methodology, and disease stage.

A particularly intriguing finding by Kamiya et al. complicates the traditionally immunosuppressive narrative of *IL-35*. Their in vitro study showed that *IL-35*, in combination with RANKL, enhanced osteoclastogenesis via ERK and MAPK signaling pathways—mechanisms central to bone resorption [72]. This suggests *IL-35* may, under certain microenvironmental conditions, contribute to tissue destruction rather than protection [72]. Such duality mirrors context-dependent behaviors observed in systemic diseases and underlines the need to explore *IL-35*’s role in specific cellular and cytokine milieus.

From a systems-level perspective, Eriksson et al. highlighted the significance of *IL-35* in patients with comorbid RA and periodontitis, where it was among several cytokines elevated in response to distinct microbiome inflammation signatures [69]. This finding underscores *IL-35*’s broader immunological role in the convergence of oral and systemic inflammatory diseases and advocates for integrative biomarker strategies.

Overall, current data portray *IL-35* as a pleiotropic and context-sensitive cytokine, with evidence of both protective and pathogenic potential in periodontitis. While its local elevation is generally interpreted as an anti-inflammatory feedback response, recent findings suggest that under certain conditions, *IL-35* may participate in bone resorption and tissue remodeling. In the context of periodontitis, *IL-35* production by regulatory B cells (Bregs) is critically dependent on signals from pro-resolving M2 macrophages, specifically via the PD-L1/PD-1 pathway [91]. This study highlights a novel immunoregulatory mechanism whereby M2 macrophages, known for their anti-inflammatory roles during tissue healing, induce *IL-35*—but not *TGF-β1*—expression in Bregs through direct cell-to-cell contact and PD-L1 engagement. The selective induction of *IL-35* suggests it may serve as a key mediator in restoring immune balance in periodontal lesions, distinguishing it from other cytokines like *TGF-β1*, which appears to be regulated independently of PD-1 signaling. These findings underscore *IL-35*’s potential as a targeted immunotherapeutic agent for promoting resolution of chronic inflammation in periodontal disease [91,92].

## 9. Conclusions

*Interleukin-35* (*IL-35*) emerges as a cytokine of interest in periodontitis due to its anti-inflammatory and immunomodulatory properties. Evidence suggests that *IL-35* is locally expressed in periodontal tissues and fluids, with levels responsive to inflammation and therapy. While preliminary associations between *IL-35* gene polymorphisms (notably in *EBI3* and *IL-12A*) and disease susceptibility have been reported, findings remain inconsistent. Overall, *IL-35* holds promise as a biomarker and potential therapeutic target in periodontitis, warranting further large-scale, well-designed studies to validate its clinical relevance.

## Figures and Tables

**Table 1:** Key structural, functional, and clinical characteristics of *Interleukin-35*.

Category	Details
Cytokine Family	Heterodimer composed of *EBI3* (Epstein–Barr virus-induced gene 3) and IL-12p35 subunits
Primary Cellular Sources	Regulatory T cells (CD4^+^ Tregs, CD8^+^ Tregs), regulatory B cells (Bregs)
Main Immunological Role	Anti-inflammatory and immunosuppressive; inhibits Th17 cell activation and IL-17 production
Key Target Cells	T helper 17 (Th17) cells, effector T cells
Mechanisms of Action	Suppresses T-cell proliferation and cytokine production—Induces *IL-35*-producing iTr35 cells—Promotes immune tolerance and resolution
Genetic Considerations	Polymorphisms in *EBI3* and *IL-12A* (p35) genes may affect cytokine expression and function
In Periodontitis	Reduced *IL-35* expression linked to increased disease severity; potential biomarker for disease activity and treatment response
In Other Diseases	Implicated in autoimmune diseases (e.g., rheumatoid arthritis, IBD), cancers, sepsis, and chronic infections
Clinical Potential	Biomarker for inflammation and immune status; therapeutic target in immune-mediated diseases

**Table 2:** Summary of Key Studies Investigating *IL-35* in Periodontitis.

Study	Focus	Model/Subjects	Main Findings	*IL-35* Role
**Jing et al.** [62]	*IL-35* from plasma cells in CP	Human CP tissues	Identified *IL-35*/*IL-37*-producing plasma cells; inhibited osteoclastogenesis	Anti-inflammatory, bone-protective
**Han et al.** [63]	CD25^+^ Bregs in periodontitis	Murine model, B cells	*IL-35* and *TGF-β* from Bregs reduced bone loss and modulated Th-cell balance	Protective, regulatory
**Hassan et al.** [64]	*IL-35*/*IL-39* in diabetic and non-diabetic CP	38 patients	*IL-35* decreased in diabetics; increased after therapy	Potential diagnostic/prognostic biomarker
**Durga et al.** [65]	*IL-35* SNPs and susceptibility (±T2DM)	96 participants	No significant SNP association found	Negative genetic evidence
**Maboudi et al.** [66]	Serum *IL-35* in CP and diabetes	72 participants	No significant serum differences; local relevance suspected	Unlikely systemic biomarker
**Taskaldiran et al.** [67]	*IL-35* in smokers vs. non-smokers with CP	57 total (3 groups)	Lower *IL-35* in smokers; higher *IL-35* in non-smokers with CP	Modulated by smoking
**Jin et al.** [68]	*IL-35* in PBMCs, GCF, and tissues in CP	Human samples	Elevated *IL-35* *mRNA*/protein in CP; inverse correlation with disease severity	Protective, locally active
**Eriksson et al.** [69]	*IL-35* in RA + CP comorbidity	101 subjects	*IL-35* elevated in comorbid patients; correlated with microbial profiles	Immune-modulating, systemic marker
**Mitani et al.** [24]	*IL-35* vs. IL-17 in GCF and tissue	Periodontitis vs. healthy	*IL-35* correlated with PD, CAL; IL-27 not detectable	Anti-inflammatory counterbalance to IL-17
**Ho et al.** [70]	*IL-35* in GCF vs. plasma across health/disease	Healthy, gingivitis, periodontitis	Plasma *IL-35* increased with disease severity	Systemic response marker
**Altaca et al.** [71]	*IL-35*, IL-6, IL-17 in GCF	90 participants	*IL-35* elevated in advanced disease; associated but not correlated with clinical indices	Disease presence biomarker
**Kamiya et al.** [72]	*IL-35* effects on osteoclastogenesis	RAW264.7 murine cells	*IL-35* enhanced bone resorption with RANKL, ERK activation	Dual role: context-dependent
**Köseoğlu et al.** [73]	*IL-35* in GCF, saliva, and plasma	60 participants	GCF *IL-35* high in disease (total), but higher concentration in health	Anti-inflammatory, concentration-sensitive
**Cafferata et al.** [53]	*IL-35* treatment in murine periodontitis	Mouse model	Reduced bone loss via Th17/Treg modulation	Therapeutic candidate

## Data Availability

No new data were created or analyzed in this study. Data sharing is not applicable to this article.

## Abbreviations

The following abbreviations are used in this manuscript:

IL-35	Interleukin-35
IL-1β	Interleukin-1β
TNF-α	Tumor necrosis factor-alpha
IL-6	Interleukin-6
MMP	Matrix metalloproteinase
IL-1Ra	Interleukin-1 receptor antagonist
TIMPs	Tissue inhibitors of metalloproteinases
SNPs	Single-nucleotide polymorphisms
IL-1	Interleukin-1
IL-6	Interleukin-6 (duplicate)
TNF-α	Tumor necrosis factor-alpha (duplicate)
TLR	Toll-like receptor
IL-12	Interleukin-12
Bregs	Regulatory B cells
CD4^+^ Tregs	CD4^+^ regulatory T cells
iTr35	IL-35-induced regulatory T cells
Th1	T helper 1
NSCLC	Non-small-cell lung cancer
RA	Rheumatoid arthritis
SLE	Systemic lupus erythematosus
HCV	Hepatitis C virus
COPD	Chronic obstructive pulmonary disease
CAD	Coronary artery disease
VKH	Vogt–Koyanagi–Harada syndrome
IFN-γ	Interferon gamma
TLR3	Toll-like receptor 3
TLR4	Toll-like receptor 4
T2DM	Type 2 diabetes mellitus
GCF	Gingival crevicular fluid
RFLP	Restriction fragment length polymorphism
IL-23	Interleukin-23
FBS	Fasting blood sugar
HbA1c	Hemoglobin A1c
ESR	Erythrocyte sedimentation rate
CRP	C-reactive protein
ELISA	Enzyme-linked immunosorbent assay
CAL	Clinical attachment loss
GCF	Gingival crevicular fluid (duplicate)
PD	Probing depth
RANKL	Receptor activator of NF-κB ligand
TRAP	Tartrate-resistant acid phosphatase
ERK	Extracellular signal-regulated kinase
MAPK	p38 mitogen-activated protein kinase
PI	Plaque index
micro-CT	Micro-computed tomography
NSPT	Non-surgical periodontal therapy

## References

[1] Kozak M., Dabrowska-Zamojcin E., Mazurek-Mochol M., Pawlik A. (2020). Cytokines and their genetic polymorphisms related to periodontal disease. J. Clin. Med..

[2] Otenio C., Fonseca I., Martins M., Ribeiro L., Assis N., Ferreira A., Ribeiro R. (2012). Expression of IL-1, IL-6, TNF-α, and iNOS in pregnant women with periodontal disease. Genet. Mol. Res..

[3] Wong H.C., Ooi Y., Pulikkotil S.J., Naing C. (2018). The role of three interleukin 10 gene polymorphisms (−1082 A > G, −819 C > T, −592 A > C) in the risk of chronic and aggressive periodontal disease: A meta-analysis and trial sequential analysis. BMC Oral Health.

[4] Johnson G.K., Guthmiller J.M. (2007). The impact of cigarette smoking on periodontal disease and treatment. Periodontology.

[5] Mira A., Simon-Soro A., Curtis M.A. (2017). Role of microbial communities in the pathogenesis of periodontal diseases and caries. J. Clin. Periodontol..

[6] Yuvashri P., Devi R.R., Nalini H.E., Prasad P.A.K. (2024). Estimating the salivary levels of IL-35 in smokers with periodontitis: A cross-sectional study. Saudi Dent. J..

[7] World Health Organization (WHO) (2017). WHO Report on the Global Tobacco Epidemic.

[8] Thomson W.M. (2014). Epidemiology of oral health conditions in older people. Gerodontology.

[9] Jin L.J., Lamster I.B., Greenspan J.S., Pitts N.B., Scully C., Warnakulasuriya S. (2016). Global burden of oral diseases: Emerging concepts, management and interplay with systemic health. Oral Dis..

[10] Darveau R.P. (2010). Periodontitis: A polymicrobial disruption of host homeostasis. Nat. Rev. Microbiol..

[11] Kassebaum N.J., Bernabe E., Dahiya M., Bhandari B., Murray C.J., Marcenes W. (2014). Global burden of severe periodontitis in 1990–2010: A systematic review and meta-regression. J. Dent. Res..

[12] Kassebaum N.J., Bernabe E., Dahiya M., Bhandari B., Murray C.J., Marcenes W. (2014). Global burden of severe tooth loss: A systematic review and meta-analysis. J. Dent. Res..

[13] Marcenes W., Kassebaum N.J., Bernabe E., Flaxman A., Naghavi M., Lopez A., Murray C.J. (2013). Global burden of oral conditions in 1990–2010: A systematic analysis. J. Dent. Res..

[14] Mawardi H.H., Elbadawi L.S., Sonis S.T. (2015). Current understanding of the relationship between periodontal and systemic diseases. Saudi Med. J..

[15] Tonetti M.S., Jepsen S., Jin L., Otomo-Corgel J. (2017). Impact of the global burden of periodontal diseases on health, nutrition and wellbeing of mankind: A call for global action. J. Clin. Periodontol..

[16] Pashova-Tasseva Z. (2022). Significance of Gene Polymorphism in Severe Periodontitis. Ph.D. Thesis.

[17] Di Benedetto A., Gigante I., Colucci S., Grano M. (2013). Periodontal disease: Linking the primary inflammation to bone loss. Clin. Dev. Immunol..

[18] Pérez-Rubio G., López-Flores L., Ramírez-Venegas A., Noé-Díaz V., García-Gómez L., Ambrocio-Ortiz E., Sánchez-Romero C., Hernández-Zenteno R.J., Sansores R.H., Falfán-Valencia R. (2017). Genetic polymorphisms in CYP2A6 are associated with a risk of cigarette smoking and predispose to smoking at younger ages. Gene.

[19] Ksiazek K., Blaszczak J., Buraczynska M. (2019). IL4 gene VNTR polymorphism in chronic periodontal disease in end-stage renal disease patients. Oral Dis..

[20] Olson B.M., Jankowska-Gan E., Becker J.T., Vignali D.A., Burlingham W.J., McNeel D.G. (2012). Human prostate tumor antigen-specific CD8+ regulatory T cells are inhibited by CTLA-4 or IL-35 blockade. J. Immunol..

[21] Okada K., Fujimura T., Kikuchi T., Aino M., Kamiya Y., Izawa A., Iwamura Y., Goto H., Okabe I., Miyake E. (2017). Effect of interleukin (IL)-35 on IL-17 expression and production by human CD4+ T cells. PeerJ.

[22] Cai S., Liu X., Li H., Feng L., Cao L., Huang J., Liang Y., Zeng J., Shi B. (2017). Elevated interleukin (IL)-35 related sCD14 but not IL-23 is associated with the severity of chronic periodontitis. Int. J. Clin. Exp. Med..

[23] Schmidlin P.R., Dehghannejad M., Fakheran O. (2021). Interleukin-35 pathobiology in periodontal disease: A systematic scoping review. BMC Oral Health.

[24] Mitani A., Niedbala W., Fujimura T., Mogi M., Miyamae S., Higuchi N., Abe A., Hishikawa T., Mizutani M., Ishihara Y. (2015). Increased expression of interleukin (IL)-35 and IL-17, but not IL-27, in gingival tissues with chronic periodontal disease. J. Periodontol..

[25] Omenesa Bello R., Chin V.K., Abd Rachman Isnadi M.F., Abd Majid R., Atmadini Abdullah M., Lee T.Y., Amiruddin Zakaria Z., Hussain M.K., Basir R. (2018). The role, involvement and function(s) of interleukin-35 and interleukin-37 in disease pathogenesis. Int. J. Mol. Sci..

[26] Yoshimoto T. (2013). Introduction. Cytokine Frontiers: Regulation of Immune Responses in Health and Disease.

[27] Ye C., Yano H., Workman C.J., Vignali D.A. (2021). Interleukin-35: Structure, function and its impact on immune-related diseases. J. Interferon Cytokine Res..

[28] Vignali D.A., Kuchroo V.K. (2012). IL-12 family cytokines: Immunological playmakers. Nat. Immunol..

[29] Choi J., Leung P.S., Bowlus C., Gershwin M.E. (2015). IL-35 and autoimmunity: A comprehensive perspective. Clin. Rev. Allergy Immunol..

[30] Hao S., Chen X., Wang F., Shao Q., Liu J., Zhao H., Yuan C., Ren H., Mao H. (2018). Breast cancer cell-derived IL-35 promotes tumor progression via induction of IL-35-producing induced regulatory T cells. Carcinogenesis.

[31] Tewari N., Zaitoun A.M., Arora A., Madhusudan S., Ilyas M., Lobo D.N. (2013). The presence of tumour-associated lymphocytes confers a good prognosis in pancreatic ductal adenocarcinoma: An immunohistochemical study of tissue microarrays. BMC Cancer.

[32] Pylayeva-Gupta Y., Das S., Handler J.S., Hajdu C.H., Coffre M., Koralov S.B., Bar-Sagi D. (2016). IL-35-producing B cells promote the development of pancreatic neoplasia. Cancer Discov..

[33] Nishino R., Takano A., Oshita H., Ishikawa N., Akiyama H., Ito H., Nakayama H., Miyagi Y., Tsuchiya E., Kohno N. (2011). Identification of Epstein–Barr virus-induced gene 3 as a novel serum and tissue biomarker and a therapeutic target for lung cancer. Clin. Cancer Res..

[34] Zhang T., Nie J., Liu X., Han Z., Ding N., Gai K., Liu Y., Chen L., Guo C. (2021). Correlation analysis among the level of IL-35, microvessel density, lymphatic vessel density, and prognosis in non-small cell lung cancer. Clin. Transl. Sci..

[35] Nakano S., Morimoto S., Suzuki S., Tsushima H., Yamanaka K., Sekigawa I., Takasaki Y. (2015). Immunoregulatory role of IL-35 in T cells of patients with rheumatoid arthritis. Rheumatology.

[36] Ning X., Jian Z., Wang W. (2015). Low serum levels of interleukin-35 in patients with rheumatoid arthritis. Tohoku J. Exp. Med..

[37] Li Y., Yao L., Liu S., Wu J., Xia L., Shen H., Lu J. (2019). Elevated serum IL-35 levels in rheumatoid arthritis are associated with disease activity. J. Investig. Med..

[38] Ye Z., Jiang Y., Sun D., Zhong W., Zhao L., Jiang Z. (2019). The plasma interleukin (IL)-35 level and frequency of circulating IL-35(+) regulatory B cells are decreased in a cohort of Chinese patients with new-onset systemic lupus erythematosus. Sci. Rep..

[39] Guan S.Y., Liu L.N., Mao Y.M., Zhao C.N., Wu Q., Dan Y.L., Pan H.F. (2019). Association between interleukin-35 gene single nucleotide polymorphisms and systemic lupus erythematosus in a Chinese Han population. Biomolecules.

[40] Singh K., Martinell M., Luo Z., Espes D., Stålhammar J., Sandler S., Carlsson P.O. (2019). Cellular immunological changes in patients with LADA are a mixture of those seen in patients with type 1 and type 2 diabetes. Clin. Exp. Immunol..

[41] Lin Y., Huang Y., Lu Z., Luo C., Shi Y., Zeng Q., Lin Y., Huang Y., Lu Z., Luo C. (2011). Decreased plasma IL-35 levels are related to the left ventricular ejection fraction in coronary artery diseases. PLoS ONE.

[42] Liu F., Tong F., He Y., Liu H. (2011). Detectable expression of IL-35 in CD4+ T cells from peripheral blood of chronic hepatitis B patients. Clin. Immunol..

[43] Liu Y., Luo Y., Zhu T., Jiang M., Tian Z., Tang G., Liang X. (2021). Regulatory B cells dysregulated T cell function in an IL-35-dependent way in patients with chronic hepatitis B. Front. Immunol..

[44] Zhou Y., Zhang H., Li Y. (2015). IL-35 expression in peripheral blood CD4(+) T cells from chronic hepatitis B virus-infected patients directly correlates with virus load. Cytokine.

[45] Liu S., Zhang Q., Shao X., Wang W., Zhang C., Jin Z. (2017). An immunosuppressive function of interleukin-35 in chronic hepatitis C virus infection. Int. Immunopharmacol..

[46] Jiang S., Shan F., Zhang Y., Jiang L., Cheng Z. (2018). Increased serum IL-17 and decreased serum IL-10 and IL-35 levels correlate with the progression of COPD. Int. J. Chron. Obstruct. Pulmon. Dis..

[47] Posadas-Sánchez R., Pérez-Hernández N., Angeles-Martínez J., López-Bautista F., Villarreal-Molina T., Rodríguez-Pérez J.M., Vargas-Alarcón G. (2017). Interleukin-35 polymorphisms are associated with decreased risk of premature coronary artery disease, metabolic parameters, and IL-35 levels: The Genetics of Atherosclerotic Disease (GEA) study. Mediat. Inflamm..

[48] Feng M., Zhou S., Liu T., Yu Y., Su Q., Li X., Lin W. (2021). Association between interleukin-35 gene single nucleotide polymorphisms and the uveitis immune status in a Chinese Han population. Front. Immunol..

[49] Kalburgi N.B., Muley A., Shivaprasad B.M., Koregol A.C. (2013). Expression profile of IL-35 mRNA in gingiva of chronic periodontitis and aggressive periodontitis patients: A semiquantitative RT-PCR study. Dis. Markers.

[50] Goriely S., Molle C., Nguyen M., Albarani V., Haddou N.O., Lin R., De Wit D., Flamand V., Willems F., Goldman M. (2006). Interferon regulatory factor 3 is involved in Toll-like receptor 4 (TLR4)- and TLR3-induced IL-12p35 gene activation. Blood.

[51] Ohkura N., Kitagawa Y., Sakaguchi S. (2013). Development and maintenance of regulatory T cells. Immunity.

[52] Shindo S., Hosokawa Y., Hosokawa I., Shiba H. (2019). Interleukin (IL)-35 suppresses IL-6 and IL-8 production in IL-17A-stimulated human periodontal ligament cells. Inflammation.

[53] Cafferata E.A., Terraza-Aguirre C., Barrera R., Faúndez N., González N., Rojas C., Melgar-Rodríguez S., Hernández M., Carvajal P., Cortez C. (2020). Interleukin-35 inhibits alveolar bone resorption by modulating the Th17/Treg imbalance during periodontitis. J. Clin. Periodontol..

[54] Wu H., Li P., Shao N., Ma J., Ji M., Sun X., Wu H., Li P., Shao N., Ma J. (2012). Aberrant expression of Treg-associated cytokine IL-35 along with IL-10 and TGF-beta in acute myeloid leukemia. Oncol. Lett..

[55] Whitehead G.S., Wilson R.H., Nakano K., Burch L.H., Nakano H., Cook D.N. (2012). IL-35 production by inducible costimulator (ICOS)-positive regulatory T cells reverses established IL-17-dependent allergic airways disease. J. Allergy Clin. Immunol..

[56] Collison L.W., Chaturvedi V., Henderson A.L., Giacomin P.R., Guy C., Bankoti J., Finkelstein D., Forbes K., Workman C.J., Brown S.A. (2010). IL-35-mediated induction of a potent regulatory T cell population. Nat. Immunol..

[57] Bai J., Qiu S.L., Zhong X.N., Huang Q.P., He Z.Y., Zhang J.Q., Liu G.N., Li M.H., Deng J.M. (2012). Erythromycin enhances CD4+ Foxp3+ regulatory T-cell responses in a rat model of smoke-induced lung inflammation. Mediat. Inflamm..

[58] Li X., Mai J., Virtue A., Yin Y., Gong R., Sha X., Gutchigian S., Frisch A., Hodge I., Jiang X. (2012). IL-35 is a novel responsive anti-inflammatory cytokine—A new system of categorizing anti-inflammatory cytokines. PLoS ONE.

[59] Kaustubh T.S., Manohar B.L., Charde P., Jaiswal P. (2017). Comparative evaluation of interleukin-35 levels in gingival crevicular fluid in patients with chronic gingivitis and chronic periodontitis. Int. J. Oral Care Res..

[60] Bassagh A., Hayatbakhsh Abasi M., Larussa T., Ghazizadeh M., Nemati M., Mirkamandar E., Jafarzadeh A. (2018). Diminished circulating concentration of interleukin-35 in Helicobacter pylori-infected patients with peptic ulcer: Its association with FOXP3 gene polymorphism, bacterial virulence factor CagA, and gender of patients. Helicobacter.

[61] Barnes V., Kennedy A., Panagakos F., Devizio W., Trivedi. H., Jönsson. T., Guo L., Cervi S., Scannapieco F. (2014). Global metabolomic analysis of human saliva and plasma from healthy and diabetic subjects, with and without periodontal disease. PLoS ONE.

[62] Xie Q., Xu W.D., Pan M., Lan Y.Y., Liu X.Y., Su L.C., Huang A.F. (2021). Association of IL-35 expression and gene polymorphisms in rheumatoid arthritis. Int. Immunopharmacol..

[63] Jing L., Kim S., Sun L., Wang L., Mildner E., Divaris K., Jiao Y., Offenbacher S. (2019). IL-37- and IL-35/IL-37-producing plasma cells in chronic periodontitis. J. Dent. Res..

[64] Han Y., Yu C., Yu Y., Bi L. (2022). CD25+ B cells produced IL-35 and alleviated local inflammation during experimental periodontitis. Oral Dis..

[65] Hassan S.S., Abdelkawy M., Shaker O.G., Tarrad N.A.F. (2024). IL-39 and IL-35 gingival crevicular fluid levels in diabetic patients with generalized periodontitis. Clin. Oral Investig..

[66] Durga J.S.V., Devi R.R., Sonika S., Vignesh T. (2024). Assessment of IL-35 gene polymorphism in periodontitis patients with and without diabetes: A cross-sectional study. J. Pharm. Bioallied Sci..

[67] Maboudi A., Eghbalian-Nouzanizadeh A., Seifi H., Bahar A., Mohadese M., Mohammadpour A., Abediankenari S., Poorbaghi S.L., Sepehrimanesh M. (2019). Serum levels of interleukin-23 and -35 in patients with and without type 2 diabetes mellitus and chronic periodontitis. Caspian J. Intern. Med..

[68] Taskaldiran E.S., Tuter G., Yucel A.A., Yaman M. (2024). Effects of smoking on the salivary and GCF levels of IL-17 and IL-35 in periodontitis. Odontology.

[69] Jin Y., Liu D., Lin X. (2017). IL-35 may maintain homeostasis of the immune microenvironment in periodontitis. Exp. Ther. Med..

[70] Eriksson K., Lundmark A., Delgado L.F., Hu Y.O.O., Fei G., Lee L., Fei C., Catrina A.I., Jansson L., Andersson A.F. (2022). Salivary Microbiota and Host-Inflammatory Responses in Periodontitis Affected Individuals with and Without Rheumatoid Arthritis. Front. Cell Infect. Microbiol..

[71] Ho J.Y., Yeo B.S., Yang X.L., Thirugnanam T., Hakeem M.F., Sahu P.S., Pulikkotil S.J. (2021). Local and Systemic Expression Profile of IL-10, IL-17, IL-27, IL-35, and IL-37 in Periodontal Diseases: A Cross-sectional Study. J. Contemp. Dent. Pract..

[72] Altaca M., Cebesoy E.I., Kocak-Oztug N.A., Bingül I., Cifcibasi E. (2024). Interleukin-6, -17, and -35 levels in association with clinical status in stage III and stage IV periodontitis: A cross-sectional study. BMC Oral Health.

[73] Kamiya Y., Kikuchi T., Goto H., Okabe I., Takayanagi Y., Suzuki Y., Sawada N., Okabe T., Suzuki Y., Kondo S. (2020). IL-35 and RANKL Synergistically Induce Osteoclastogenesis in RAW264 Mouse Monocytic Cells. Int. J. Mol. Sci..

[74] Köseoğlu S., Sağlam M., Pekbağrıyanık T., Savran L., Sütçü R. (2015). Level of Interleukin-35 in Gingival Crevicular Fluid, Saliva, and Plasma in Periodontal Disease and Health. J. Periodontol..

[75] Raj S.C., Panda S.M., Dash M., Patnaik K., Mohanty D., Katti N., Mahapatra A., Mishra D., Praharaj K. (2018). Association of Human Interleukin-35 Level in Gingival Crevicular Fluid and Serum in Periodontal Health, Disease, and after Nonsurgical Therapy: A Comparative Study. Contemp. Clin. Dent..

[76] Goswamy A., Hans M., Hans V.M., Sheokand V., Grover H.S. (2022). Effect of nonsurgical periodontal therapy on gingival crevicular fluid levels of Interleukin-35 in patients with periodontitis. J. Oral Biol. Craniofac. Res..

[77] Durgapal S., Shetty M. (2025). Effect of non-surgical periodontal therapy on the salivary levels of IL-18 and IL-35 in patients with periodontitis. Dent. Med. Probl..

[78] Jadhav A., Rathod S., Kolte A. Levels of Interleukin-35 Changes in Gingival Crevicular Fluid After Non-Surgical Periodontal Therapy in Stage III Periodontitis: A Randomized, Controlled Clinical Trial. https://www.researchgate.net/publication/361044830_Levels_of_Interleukin-35_changes_in_Gingival_Crevicular_Fluid_after_Non-Surgical_Periodontal_Therapy_in_stage_III_periodontitis_a_randomized_controlled_clinical_Trial.

[79] Thakare K.S., Charde P.A., Bhongade M.L., Jaiswal P.P., Bajaj P.S. (2022). Evaluation of gingival crevicular fluid interleukin-35 as a marker for identification of periodontal disease activity. Minerva Dent. Oral Sci..

[80] Rakic M., Pejcic N., Perunovic N., Vojvodic D. (2021). A roadmap towards precision periodontics. Medicina.

[81] Gundelly M., Pusuluri S.V., Koduganti R.R., Ambati M., Chiluveru S., Chandaka M., Mrunalini G., Pusuluri S., Sneha C., Chandaka M. (2024). Precision Medicine in Periodontics: A Literature Review. Cureus.

[82] Suwinski P., Ong C., Ling M.H.T., Poh Y.M., Khan A.M., Ong H.S. (2019). Advancing Personalized Medicine through the Application of Whole Exome Sequencing and Big Data Analytics. Front. Genet..

[83] Stefanicka-Wojtas D., Kurpas D. (2023). Personalised Medicine—Implementation to the Healthcare System in Europe (Focus Group Discussions). J. Pers. Med..

[84] Rai B., Kharb S., Jain R., Anand S.C. (2008). Biomarkers of periodontitis in oral fluids. J. Oral Sci..

[85] Schulz S., Stein J.M., Altermann W., Klapproth J., Zimmermann U., Reichert Y., Gläser C., Schaller H.G., Reichert S. (2011). Single nucleotide polymorphisms in interleukin-1 gene cluster and subgingival colonization with *Aggregatibacter actinomycetemcomitans* in patients with aggressive periodontitis. Hum. Immunol..

[86] Kornman K.S., Page R.C., Tonetti M.S. (1997). The host response to the microbial challenge in periodontitis: Assembling the players. Periodontol. 2000.

[87] Cui X., Liu W., Jiang H., Zhao Q., Hu Y., Tang X., Liu X., Dai H., Rui H., Liu B. (2024). IL-12 family cytokines and autoimmune diseases: A potential therapeutic target?. J. Transl. Autoimmun..

[88] Bakery H.H., Hussein H.A.A., Ahmed O.M., Abuelsaad A.S.A., Khalil R.G. (2024). The potential therapeutic role of IL-35 in pathophysiological processes in type 1 diabetes mellitus. Cytokine.

[89] Chakraborty R., Mukherjee A.K., Bala A. (2024). Breakthroughs in road mapping IL-35 mediated immunotherapy for type-1 and autoimmune diabetes mellitus. Cytokine.

[90] Ezzeddini R., Somi M.H., Taghikhani M., Moaddab S.Y., Masnadi Shirazi K., Shirmohammadi M., Eftekharsadat A.T., Sadighi Moghaddam B., Salek Farrokhi A. (2021). Association of Foxp3 rs3761548 polymorphism with cytokines concentration in gastric adenocarcinoma patients. Cytokine.

[91] Goto H., Kikuchi T., Takayanagi Y., Kamiya Y., Suzuki Y., Kawamura S., Sawada N., Hayashi J.I., Mitani A. (2023). Ebi3 knockout aggravates experimental periodontitis via Th17 polarization. J. Clin. Periodontol..

[92] Cao G., Memida T., Huang S., Dalir Abdolahinia E., Ruiz S., Hassantash S., Ari J., Shindo S., Lin J., Kawai T. (2025). Pro-Resolving Macrophage-Induced IL-35+ but Not TGF-β1+ Regulatory B Cell Activation Requires the PD-L1/PD-1 Pathway. Int. J. Mol. Sci..

